# Quantitative histological image analyses of reticulin fibers in a myelofibrotic mouse

**DOI:** 10.14440/jbm.2016.152

**Published:** 2016-11-22

**Authors:** Hector A. Lucero, Shenia Patterson, Shinobu Matsuura, Katya Ravid

**Affiliations:** Department of Medicine, Whitaker Cardiovascular Institute, Boston University School of Medicine, Boston, MA 02118, USA

**Keywords:** reticulin fibrosis, image analysis, ImageJ, Gata-1low mice

## Abstract

Bone marrow (BM) reticulin fibrosis (RF), revealed by silver staining of tissue sections, is associated with myeloproliferative neoplasms, while tools for quantitative assessment of reticulin deposition throughout a femur BM are still in need. Here, we present such a method, allowing *via* analysis of hundreds of composite images to identify a patchy nature of RF throughout the BM during disease progression in a mouse model of myelofibrosis. To this end, initial conversion of silver stained BM color images into binary images identified two limitations: variable color, owing to polychromatic staining of reticulin fibers, and variable background in different sections of the same batch, limiting application of the color deconvolution method, and use of constant threshold, respectively. By blind coding image identities, to allow for threshold input (still within a narrow range), and using shape filtering to further eliminate background we were able to quantitate RF in myelofibrotic Gata-1low (experimental) and wild type (control) mice as a function of animal age. Color images spanning the whole femur BM were batch-analyzed using ImageJ software, aided by our two newly added macros. The results show heterogeneous RF density in different areas of the marrow of Gata-1low mice, with degrees of heterogeneity reduced upon aging. This method can be applied uniformly across laboratories in studies assessing RF remodeling induced by aging or other conditions in animal models.

## INTRODUCTION

Myeloproliferative neoplasms (MPN) include polycythemia vera, essential thrombocythemia, and primary myelofibrosis (PMF), as observed in clinical and bone marrow (BM) morphologic findings [1]. A number of mutations have been described in this group of diseases [2-5], as well as disorders in Gata-1 transcription factor [6, 7]. A common feature in several MPN forms, and particularly in PMF is the abnormal presence of abundant reticulin fibers in the BM. Mouse models of PMF, including JAK2V617F [8, 9] and GATA-1low mice [6, 10] have significant hallmarks of human pathology.

Silver impregnation to visualize subcellular structures was the keystone method used by Santiago Ramón y Cajal and Camillo Golgi, leading to their 1906 shared Nobel Prize in Physiology or Medicine. By 1937 György Gömöri consolidated the silver impregnation technique to visualize tissue reticulin fibers [11]. Since then, the method has been applied to a variety of pathological onsets, particularly in the clinical assessment of the BM fibrotic degeneration dynamics [12]. In the case of human pathology, the BM trephine biopsy is graded on a semi-quantitative scale [12] distinguished by the number, density, thickness of reticulin fibers, and the development of collagen fibrosis, as assessed by experienced pathologists. Increased reticulin grade is regarded as an adverse prognostic factor in myeloproliferative neoplasms [13]. A computer assisted RF analysis, using color deconvolution showed a general correlation with the semi-quantitative grading methods [14]. However, the use of color deconvolution analysis is hampered since silver impregnation does not yield monochromatic reticulin fibers.

Assessment of BM fibrosis in mouse models has relied on using reticulin staining followed by gross evaluation of density visually or semi-quantitatively analyzed. The need for a uniform and quantitative approach across laboratories to measuring fibrotic burden is eminent when the process has to be followed as function of age, or comparatively between experimental models or conditions. Here, we describe a method of quantification of RF, overcoming two main limitations associated with image analysis: (1) variable color of the silver impregnated reticulin fibers; and (2) variable background staining. This method is not meant to replace the broadly used and accepted, though not exempt of problems and pitfalls [15], subjective grading method used by pathologists. Rather, it is fit to assess progression of RF throughout bone marrow femurs of animal model models. Our quantitative approach revealed a heterogeneous distribution of RF throughout bone marrow of a myelofibrotic mouse, the dynamics of which changes upon disease progression.

## MATERIALS AND METHODS

### Animals, histology and Gömöri's silver impregnation

A comprehensive study described phenotype variation in GATA-1low mice depending on genetic background [16]. Here, we used GATA-1low mice (ΔneoΔHS) originating from [17], and available as mixed 129/ C57BL/6 background [18]. These GATA-1low mice reach adulthood and develop bone marrow fibrosis, compared to none in matching controls, as we also observed before [19]. Mouse femurs were isolated, fixed and sent for tissue sectioning and slide mounting by AML laboratories (http://www.amllabs.com/contactus.html), following conventional protocols. Hematoxylin and Eosin staining of the cells and nuclei, and Gömöri's silver impregnation of reticulin fibers were carried out as described before [19]. Expansion of the mouse colonies was carried out at Boston University School of Medicine Animal Science Center. All studies involving mice were approved by the Boston University Animal Care and Use Committee.

### Method steps and main sources of variability

This quantitative image analysis is the last step in a procedure involving animal selection → femur isolation → decalcification → fixation → paraffin embedding → histological sectioning → slides generation → deparaffinization → silver impregnation → image acquisition → image analysis. The silver impregnation procedure, involving around 20 steps and the use of 9 chemicals is an important source of analytical variability. However, several refinements of the protocol have produced abundant literature to troubleshoot deficient staining outcomes.

### Femur sections arrangement in slide and sample identification

Three consecutive femurs sections (5 μm each) were placed parallel to the width of the slide and referred to as “a”, “b” and “c” from the top end. Slide number, animal number, genotype and age were recorded on the slide top end (**Fig. S1A**). Pictures, taken from each bone section (a, b, c) were numbered and the numbers were placed at the end of the picture name string. Thus, if 4 pictures were taken from section “a” in slide 8, they were saved under the following names: 3W30_8_a1, 3W30_8_a2, 3W30_8_a3, and 3W30_8_a4 (**Fig. S1B**). Hence, a large number of images, collected from multiple genotypes of animals at different ages, were unambiguously named. During image processing these annotations were coded with positive natural numbers and subsequently randomly coded with a 20 digits code as described below.

### Bone marrow imaging

Ten or more digital color (Red Green and Blue) RGB images (16000 × 12000 pixels) per stained and mounted bone sections were captured along the diaphysis longitudinal axis in a Nikon Eclipse 50i microscope (20 × /0.40 objective and ocular 10 × /22) with a color digital camera (Diagnostic Instruments, Color Mosaic 18.2) controlled by the Spot Image software v5.0.37. TIFF images were saved to a dedicated folder and their filenames recorded in an Excel sheet. The original file names, organized by slide numbers within each genotype, were coded by a single number following the list order. Then the corresponding image folder was blind-coded using the broadly applicable routine (BAR v1.1.6) for ImageJ. The folder was processed in ImageJ (BAR → Snippets → Process Folder ImageJ) to generate a new folder containing image files with their names blinded to a 20 digits name (**Table S1**).

### Determination of actual bone marrow area occupancy

Using ImageJ platform [20], color images are cleared of artifacts (bone sections, out of focus areas and dark spots) and the actual BM area is measured by selecting the BM contour with the free-hand selection tool and then executing: Analyze → Set Measurements → Select only “Area” and “Display label” → Measure. The “Results” table will record the image name and the area corresponding to the BM. The maximal BM area will be equal to the whole image area, 1600 × 1200 = 1920000 pixels. The ratio, “Total image area”/“BM area” renders a factor ≥ 1.0 that is later used to normalize the area occupied by reticulin fibers with respect to the total image area (**Fig. S2**). This procedure can be batch processed by running an appropriate macro, which we added to the application (**Script S1**).

### Acquisition and processing of whole femur images

This procedure involves few distinct steps: (1) Overlapping pictures (1600 × 1200 pixels) along the whole diaphysis longitudinal axis length of the bone are taken and saved in a folder. This step generates over 200 RGB images; (2) Overlapping RGB images are stitched using Microsoft Image Composite Editor, generating a 500 MB image. The bone area in the RGB digital image is erased; (3) Change the image area unit from inches to pixel. Analyze → Set scale Type “pixels” in the “Unit of length” box. Take note of the width and length values shown in the upper left corner of the image (*i.e.*, 13344 × 4920 pixels). Divide 13344/1600 = 8.34 and 4920/1200 = 4.1; (4) Fragment the large image into smaller images to facilitate image cleaning in next step: Open the whole BM image → Image → Stacks → Tools → Montage to Stacks. In the Stack Maker window, select Images per row = 8 and Images per column = 4. This mode of fragmentation generates 32 images of a manageable size, close to the size of original ones that were stitched to produce the large image; (5) Erase image artifacts in the stack using the Paintbrush Tool with a width set at around 30 pixels; (6) Get the 32 images from the stack: Image → Stacks → Stack to Images; and (7) Save the RGB images in a dedicated folder for further analysis.

### Single image processing

Corrected RGB images are subjected to the following procedure: (1) Convert to RGB Stacks Image → Type → RGB Stacks; (2) Image → Color → Arrange Channels → Select only “2” in the “New channel order” window. Accept that channels will be reduced from 3 to 1; (3) Analyze → Set Measurements → Check only “Integrated density” (IntDen), “Limit to threshold” and “Display label” options. The “Display label” mode associates the file name with the corresponding IntDen value, critical for final data compilation and presentation; (4) Image → Adjust → Threshold → Set the upper slider to zero and adjust the lower slider till only fiber structures (highlighted in red) remain → Measure. **Note**: In the Results table the useful columns are “Label” (file name) and “Max Thr” (threshold applied). Values recorded in “InDen” and “Raw IntDen” columns are not useful at this stage of the analysis, but are populated in step 9 below; (5) Activate “Apply” in the Threshold window to get “Convert to Mask” the thresholded image; (6) Edit → Invert the image. Required for Shape Filter processing in the next step; (7) Plugins → Shape Filter → input 70–100000 in “Area”, 15-Infinite in “Feret Diameter”, and 30-Infinite in “Circularity” options → Click OK; (8) Process → Math → Divide → Input 255 to convert from a 0–255 to a 0–1 binary image; (9) Analyze → Measure. **Note**: In the Results table the useful data are now in columns “Label” (the file name) and columns “InDen”/“Raw IntDen” (area of reticulin fibers); and (10) The final, processed binary image, its threshold value and its measured integrated density in the results table, are saved.

### Batch image processing

A set is the number of images to be batch-analyzed. The number can be calculated using the following formula: 10 (images per slide) × 3 (bone sections per slide) × #genotypes × #animals/genotype × #slides/animal. Thus, when analyzing 4 genotypes by using 3 animals per genotype and 2 slides per animals, the total number of images is 720 from 24 slides. The average set size analyzed in this study was around 360 images, from 2 genotypes (WT and Gata-1 low), 3 animals/genotype and 2 slides/animal. In addition, to minimize the impact of Gömöri's silver impregnation variability on the results, all slides in a set were stained at the same time.

The single image process analysis outline above was applied to sets of images in a batch process mode driven by a newly developed macro (**Script S2**). The first sub-macro calls for an “In” folder, containing the images to be analyzed and an empty “Out” folder were the binary, thresholded images will be stored. Then the Batch Command loops all images from the “In” folder to be thresholded one at the time with input from the user. The resulting thresholded images are stored in “Out” folder. The Results table, recording image blinded name and its corresponding threshold value remains open. When the first sub-macro is completed the second sub-macro calls for an “In” folder that is the “Out” folder from the first sub-macro, and an “Out” folder that will store the final binary images that were quantified for the area corresponding to reticulin fibers (integrated density). This sub-macro runs to completion without user intervention. The Results table, now containing all images files names with their corresponding threshold and integrated density values is transferred to an Excel sheet data calculations and statistical analysis (**Table S2**).

### Statistics

Statistical analysis of the data was performed by analysis of variance (ANOVA) in Microsoft Excel. Putative statistical differences among sections of a slide or among Gata-1 low age group (G20, G30 and G40) were subjected to the Tukey-Kramer test [21].

## RESULTS

We sought to develop a quantitative method that enables tailoring the software to analytical needs [22], and thus allows large batch processing towards analysis of fibrosis in hundreds of composite tissue section images covering the whole length of femur BM. This, we felt, is important, considering the known heterogeneity of fibrosis in the marrow during myelofibrosis, which is not always reflected in representative images. To achieve this goal, we resorted to the ImageJ platform as it gives the operator full access to all critical variables used in the analysis, and ability to add macros (as shown in Methods), as well as allows the reader to view and adopt such features.

The first feature we tackled was color deconvolution of RGB images from tissues or cells, which is based on the principle that the target sub-cellular component acquires a unique color after staining. However, visual inspection of Gömöri reticulin stained images often reveals fibers with color tones varying from dark purple to dark grey. To illustrate this, ImageJ color deconvolution analysis was applied to images 64_2_30_8_b2 and 64_2_30_10_a1, number coded 665 and 668 respectively (numbering procedures are explained in Methods). The region of interest was selected using three user-defined colors taken from the color of fibers, the color of the background free of objects and the color of background elements. In image 665 reticulin fibers were deconvolved almost exclusively in color 1 channel, while in image 668 reticulin fibers were deconvolved mostly in color 2 channel, and some in color 1 channel (**Fig. S3**). The polychromatic nature of RF can be directly observed by over magnification of the image (**Fig. S4**). This color variability does not affect the subjective scoring evaluation to assess the progress or remission of RF, since this approach is not quantitative and it is based on the morphology and abundance of reticulin fibers, not on their color. However, color variability is a severe limitation for the application of color deconvolution in quantitative analysis.

Image segmentation (thresholding) in quantitative analysis of image elements (shapes) requires isolation of such elements from the background. When the background is constant for all images, a single, optimal threshold value can be applied. When BM images from Gata-1 low and wild type animals were analyzed, applying the same threshold, a suboptimal output was observed in both cases: a threshold (TH) optimal to isolate reticulin fibers (TH = 152) in the Gata-1low image renders an extremely high background in the wild type image (despite their processing and staining at the same time). Conversely, the application of a threshold value optimal for the background (TH = 95) in the wild type mouse image, underestimates the reticulin fibers content in the Gata-1low image (**Fig. S5**). This calls for TH adjustment of individual images. To eliminate subjective bias in the proper assignment of threshold values, the file name (identity) of all images analyzed where assigned a random, 20 digits code name described under Materials and Methods (**Table S1**).

The ImageJ Shape Filter algorithm [23] allows for the selection of the elongated shape of reticulin fibers, distinguishable from the circular shape of nuclei, resulting in elimination of background staining of the nuclei by silver impregnation and haematoxylin (**Fig. S6**). Background exclusion in comparative analysis is particularly important in images with low content of reticulin fibers, since the impact of the background level (noise) is inversely proportional to the reticulin level (signal).

**Figure 1:**
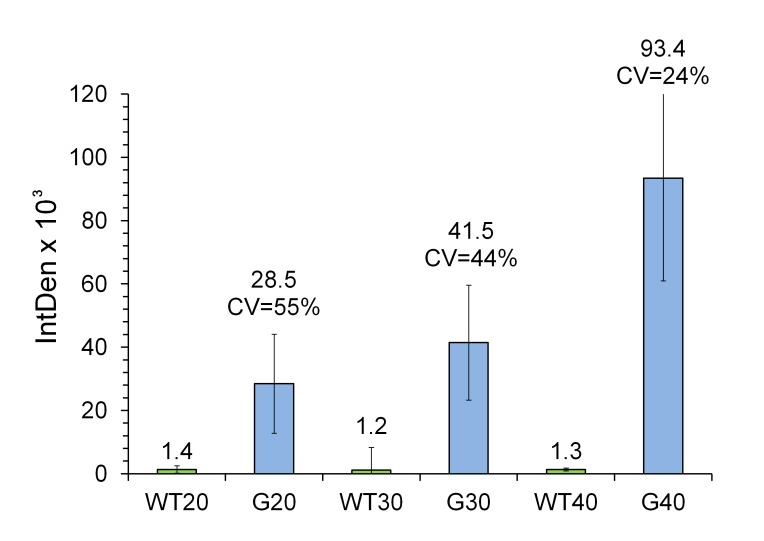
**Quantitative analysis of age related increment of reticulin fibrosis in Gata-1low mice**. Sixty images (2 slides, each with three consecutive sections) per mouse, with three animals per genotype at 20, 30 and 40 weeks old of age, totalizing 360 images, were batch processed as described under Materials and Methods. Wild type (denoted as WT) and Gata-1low (denoted as G) genotypes were analyzed. The mean integrated density (IntDen) and coefficient variance [CV = (SD/Mean) × 100] values are indicated on top of the bars. The CV indicates what percentage of the mean value is the SD. Shown are means ± SD. After rejecting the one-way ANOVA null hypothesis (*P* < 0.05) for the analysis of the 3 groups (G20, G30 and G40), the inequalities (statistical differences) among their mean values, was confirmed by applying the Tukey-Kramer multiple, pair-wise comparison test.

With a controlled quantifiable method at hand, we sought to carry out BM analysis of reticulin fibers throughout the femur BM. To this end, images of Gömöri stained sections were captured longitudinally throughout the femur, either by batch analysis of around 360 images per set, or by stitching nearly 200 overlapping images. As shown in **Figure 1**, the wild type genotype ages 20, 30 and 40 weeks old (WT20, WT30 and WT40) displayed a very low signal regardless of the age, consistent with the lack of pathological reticulin fibers in their BM. On the other hand, in Gata-1low animals (G20, G30 and G40) the reticulin content progressively increased with age, with values of mean IntDen from 28.5 × 10^3^ at 20 week-old, to 41.5 × 10^3^ at 30 week-old and, to 93.4 × 10^3^ at 40 week-old. The lower limit of detection corresponds to a 1.48% of reticulin fibers content with respect to the whole image area, as observed in G20 animals. Since no differences in reticulin content were observed among the three sections within each slide, regardless of the age or genotype (data not shown), the IntDen mean ± SD values were calculated from the 30 images captured from each slide. Coefficients of variation (CV, which indicates what percentage of the mean value is the SD) were calculated and found to be 55%, 44% and 24% for ages 20, 30 and 40 week-old, respectively.

**Figure 2:**
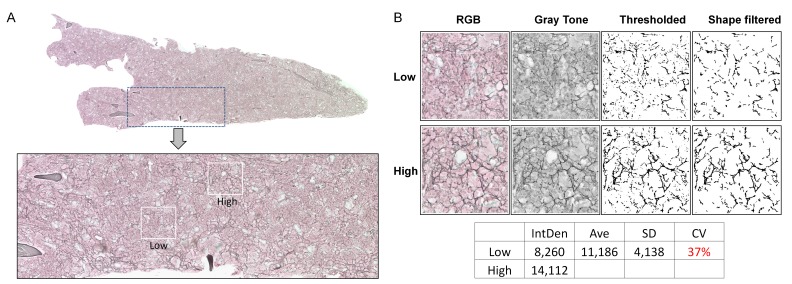
**Heterogeneous distribution of reticulin fibers within the bone marrow of Gata-1low mice**. **A**. The upper image is a composite of 208 overlapping TIFF pictures (5626 KB each) along the whole diaphysis longitudinal axis length of the bone of a 30 week-old Gata-1low mouse (representative of 3 mice analyzed). The image was captured as 20 × magnification. The bone area in the digital image was erased to reduce the image size and simplify the view. The lower image is a zoom-in of the area framed in dotted lines within the upper image, revealing areas of low and high density of fibers, framed in white boxes. The distribution of high and low intensity fibers in the bone marrow (BM) seemed not to follow a firmly recognizable localization pattern. **B**. Representative low and high density areas were extracted from the image in panel A (3 × magnification) and converted to gray scale, thresholded and shape filtered to obtain the final images for reticulin quantification. As shown in the table under the images, the coefficient of variation (CV) for the 2 values of reticulin fibrosis (low and high) in this image is 37%. The equivalent result, after analyzing nearly 360 images scattered along sections of BM from 30 weeks old Gata-1low mice yielded a CV of 44% (as in **Fig. 1**).

Considering our controlled method of detection, the above high coefficients of variation are suggested to originate from heterogeneous distribution of the fibrotic tissue within the BM. To test this possibility, we obtained composite images along the whole femur, at the same level of magnification used for the acquisition of limited BM areas as described above. This approach revealed a clear heterogeneity in the distribution of reticulin fibers within the BM (Fig. 2A). Representative high and low reticulin fiber density areas within the same specimen are shown in **Figure 2B**. This demonstrates that the high variability of the analytical data shown in **Figure 1** is mainly caused by heterogeneous distribution of reticulin fiber within the BM of a femur, which subsides in older Gata-1low mice.

**Figure 3:**
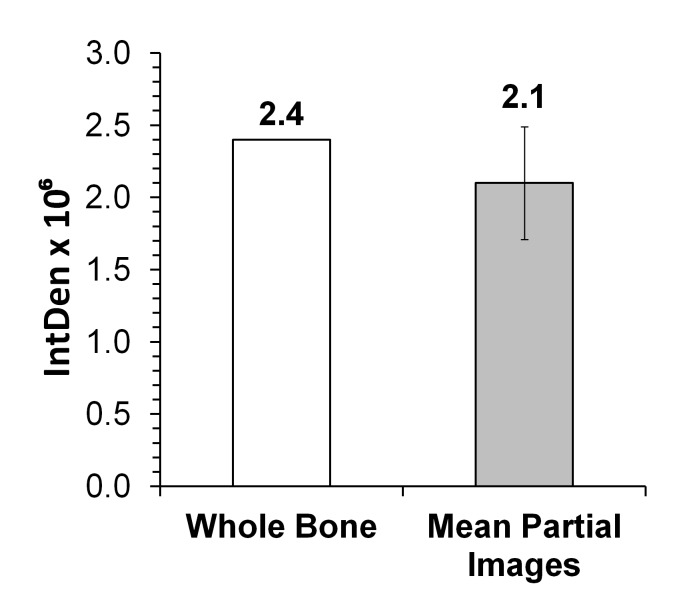
**Analysis of bone marrow section images**. Composite images, which together cover bone marrow (BM) femur length, were analyzed. A representative 30 week old Gata-1low animal shown in **Figure 2A** (empty column) and seven images of the same BM (grey shaded column) were analyzed for Integrative Intensity (IntDen). Partial images covering an area of 1.92 × 10^6^ showed a mean IntDen of 1.3 × 10^5^. This value was scaled up to the total BM image area of 31.0 × 10^6^ pixels by the following calculation: [(1.3 × 10^5^) × (31.0 × 10^6^)/1.92 × 10^6^] to obtain the IntDen 2.1 × 10^6^ (grey shaded column). The numbers on top of the columns are IntDen.

The composite analysis showed that out of 32.8 × 10^6^ pixels corresponding to the whole femur BM area, reticulin fibers occupy an area of 2.4 × 10^6^ pixels, which corresponds to 7.3% occupancy. A similar result was obtained by analysis of seven partial images of the same whole BM image, *i.e.*, reticulin fibers occupy an area of 2.1 × 10^6^ pixels, which corresponds to 6.4% occupancy (**Fig. 3**). Thus, both modes of analysis render similar results.

## DISCUSSION

Increased reticulin grade is regarded as an unfavorable prognostic factor in myelofibrotic neoplasms in human or experimental models, underscoring the need for quantitative analysis of RF progression of the disease. Computer assisted RF analysis, using color deconvolution showed a general correlation with a semi-quantitative grading methods [14]. Current protocols for evaluating BM RF include morphometric [24] and color deconvolution [14] analysis, mainly applied in human specimens. The morphometric analysis, as applied to trephine biopsies, quantitates morphological features of reticulin fibers in the context of histological, serological and clinical parameters. Color deconvolution analysis was intended to assess the total occupancy area of reticulin fibers regardless of its morphological features, but it is limited by the intrinsic polychromatic nature of silver impregnated reticulin fibers. An earlier report showed increased RF in old Gata-1low mice on CD1 background as assessed by visual and quantitative inspections of representative Gömöri stained BM sections, using the MetaMorph 6.1 Software [10, 25]. While these results continue to be informative, here, we described an imaging method focused on evaluating fibrosis *via* hundreds of composite images taken throughout bone marrow sections of femurs of a mouse model of myelofibrosis.

The new strategy presented here requires the use of a common imaging modality, within which we have developed two macros for batch processing in the steps involving: (1) image area correction; and (2) image thresholding, shape filtering and quantification. The readiness to generate macros and adjust shape filter parameters within this mode of analysis allowed us to tailor analytical needs, thus creating a platform that might be used across laboratories.

Importantly, by using the above method, we were able to determine fibrosis throughout most of the femur bone marrow, rather than in select representative sections. In the Gata-1low mice studied here, fibrosis was detected somewhat earlier and it progressed relatively slower than in Gata-1low mice on a CD1 background [10, 25]. This could be due to different mouse genetic backgrounds and method of detection. In our study we showed that reticulin deposition appears in the bone marrow as patches of different intensities, the variation of which depends on disease progression.

While image pixel occupancy of fibrosis in the marrow in the experimental model tested here is less than 10%, compared to total pixels recorded by the imaging modality, data showed that the range of heterogeneity of reticulin staining is high in younger mice (5-month, which express lower levels of overall fibrosis) than in older mice (10-month, which express more severe fibrosis). This raises the possibility that disease progression increases the percentage of areas with high fibrosis. Interestingly, whether in young or old mice, dense fibrotic patches were typically detected around sinusoids (**Fig. 2A**), which could facilitate the diffusion of circulating fibrosis-promoting factors, such as transforming growth factor-ß [25]. The known increase in vessel density in Gata-1low mice [26] could contribute to this phenotype.

In order to assess bone marrow fibrosis in humans, pathologists stain biopsy samples for reticulin and collagen fibers and grade the specimen based on a defined scale. A scale was developed by a team of international experts following evaluation of 150 paraffin-embedded biopsy sections from the posterior iliac crest of patients with bone marrow fibrosis, most of whom were diagnosed with PMF [27]. The experts, individually and without knowledge of the clinical parameters or diagnosis of the patient, graded the bone marrow biopsy sections and a consensus was achieved in follow-up meetings as a group. MF-0 (grade 0) was characterized as having a normal level of reticulin fibers that were scattered and had no intersections. Grades MF-1 to MF-4 were characterized as having gradual increases in reticulin fibers “diffuse and dense.” There was a 95% consensus in grading of the slides between pathologists with a high degree of reproducibility [27]. This method, however, requires microscopic evaluation by a highly experienced expert. It would be interesting to apply in the future the quantitative imaging method described here to specimens with pre-assigned grading by pathologists (MF-1 to MF-4), and through this comparison develop an agreed-upon quantitative imaging scale for uniform evaluation of fibrosis in human samples.

Taken together, assessing the degree of fibrotic patchiness *via* a quantitative approach would be useful in studying the dynamics of the disease in other experimental animal models, with potential diagnostic implications to human pathology, considering the limited biopsy material typically available.

## Abbreviations used

BM: bone marrow
IntDen: integrated density
MPN: myeloproliferative neoplasms
PMF: primary myelofibrosis
RF: reticulin fibrosis

## Supplementary information

**Figure S1**. Slides arrangement.

**Figure S2**. Representative image correction of a 30 week old Gata-1low male mouse, and actual bone marrow area.

**Figure S3**. Color deconvolution of Gömöri's silver impregnated bone marrow reticulin fibers.

**Figure S4**. Polychromatic nature of reticulin fibers stained by silver impregnation.

**Figure S5**. Constant *vs.* variable thresholding.

**Figure S6**. Elimination of background by Shape Filtering.

**Script S1**. Area correction macro (ImageJ macro extension).

**Script S2**. Manual threshold, shape-filter macro and quantitation (IJM extension).

**Table S1**. Correlation between image original ID and its blind-coded ID.

**Table S2**. Output data from macro in supplementary **ScriptS2**.
